# In Vitro Anticoagulant Activity and Active Components of Safflower Injection

**DOI:** 10.3390/molecules23010170

**Published:** 2018-01-15

**Authors:** Kai-Hong Wang, Shi-Fei Li, Yi Zhao, Hong-Xia Li, Li-Wei Zhang

**Affiliations:** 1 Institute of Molecular Science, Key Laboratory of Chemical Biology and Molecular Engineering of Ministry of Education, Shanxi University, Taiyuan 030006, China; wkhdfl@163.com (K.-H.W.); lisf@sxu.edu.cn (S.-F.L.); 2 Biology Institute of Shanxi, Taiyuan 030006, China; zhaoyisws@163.com (Y.Z.); lhxsws@163.com (H.-X.L.)

**Keywords:** safflower injection, anticoagulant biological activities, activated partial thromboplastin time, active component

## Abstract

Safflower injection is well-known as a traditional Chinese medicine used to improve the blood circulation. In this study, seven safflower injection samples from different companies were evaluated for their in vitro anticoagulant activity by measuring their activated partial thromboplastin time (APTT) and prothrombin time (PT) against human plasma. The screening results suggested that the safflower injections exhibited a significant prolonging influence on APTT (*p* < 0.05 vs. the control group), but not on prolonging PT (*p* > 0.05 vs. the control group). The safflower injection was separated into four fractions, and among them, fraction four demonstrated the most anticoagulant activity, with an APTT of 95.4 ± 1.4 s at a concentration of 4.0 μg/μL (*p* < 0.01 vs. control group). In addition, three active components, *p*-hydroxybenzaldehyde, *p*-hydroxy-cinnamic acid, and (8*Z*)-decaene-4,6-diyne-1-*O*-β-d-glucopyranoside were isolated from fraction four with Sephadex LH-20 and C18 column chromatography. All three active components showed significant prolonging of APTT (*p* < 0.05 vs. control group). Among them, *p*-hydroxy-cinnamic acid exhibited the most activity (*p* < 0.01 vs. control group). The results indicated that safflower injection strongly affects the intrinsic coagulation system, and we suggest that this might be the mechanism by which the safflower injection activates and promotes blood circulation.

## 1. Introduction

Cardiovascular disease can be fatal, so developing new drugs with high efficiency and low toxicity is necessary. Safflower, the dried floret of *Carthamus tinctorius*, is one of the most commonly used traditional medicines for preventing and treating cardiac disease [1]. In 2000, safflower injections were approved as a clinical therapeutic drug for the treatment of cerebral ischemia in China, and since then, have been increasingly used in clinics due to its potency and minimal side effects [2]. In general, safflower injections are prepared through the processes of decoction and alcohol precipitation. Safflower injections have been reported to display diverse pharmacological properties, such as anti-oxidation, lipid-lowering, and anti-inflammation activities. Thus, safflower is mainly used in the prevention and treatment of cardiovascular, vasculitis, occlusive cerebrovascular disease, myocardial infarction, and other diseases in clinical applications [3,4,5]. In addition, some newly published studies about the clinical effects of safflower injection combination with alprostadil and sildenafil [6], cilostazol [7] and the composite Salvia miltiorrhiza dropping pill [8] in the treatment of chronic pulmonary heart disease complicated with pulmonary hypertension, unstable angina pectoris and acute ischemic cerebral infarction have been reported. Studying the pharmacodynamic material basis of the safflower injection is necessary to ensure its safety and efficacy as a clinical medication. Many reports have been published on the clinical and pharmacological aspects of the safflower injection for hydroxysafflor yellow A (HSYA) [9,10,11,12].

Liu et al. [13] suggested that HSYA and total flavonoids were the important active ingredients in the safflower injection, proven with an in vivo test of the injection’s antithrombotic protection rate in mice, and in vitro tests of anti-platelet aggregation. Zhao et al. [14] screened out four compounds with strong anti-platelet aggregation activity, including kaempferol-3-*O*-β-rutinoside, HSYA, (8*Z*)-decaene-4,6-diyne-1-*O*-β-d-glucopyranoside, and kaempferol-3-*O*-β-sophoroside using in vitro platelet aggregation tests.

The safflower injection has been noted to significantly prolong in vitro activated partial thromboplastin time (APTT) [15]. However, to the best of our knowledge, its anticoagulant fractions and active ingredients have not yet been reported. To further investigate safflower injection’s active components and their anticoagulant mechanisms, we studied the separation and isolation of the safflower injection’s active fractions and components, as well as their ability to prolong the activated partial thromboplastin time (APTT). To clarify its anticoagulant mechanism, we also considered the prothrombin time (PT) of the safflower injection. 

## 2. Materials and Methods 

### 2.1. Safflower Injection

Safflower injections were purchased from seven different pharmaceutical companies (Shineway Pharmaceutical Co., Ltd. (Shijiazhuang, China), Shiyao Yinhu Pharmaceutical Co., Ltd. (Yuncheng, China), Huawei Pharmaceutical Co., Ltd. (Taiyuan, China), Yabao Pharmaceutical Group Co., Ltd. (Ruicheng, China), Lonch Group Wanrong Pharmaceutical Co., Ltd. (Wanrong, China), Wuhan Fuxing Bio-Pharmaceutical Co., Ltd. (Wuhan, China), Shanxi Zhengdong Ante Biological Pharmaceutical Co., Ltd. (Jinzhong, China)) (called H-1 to H-7; batch numbers in order: 16042661, 1031604204, 16071011, 170106, 17011111, 160808301, and 20170309). All safflower injections were produced in accordance with the criteria of National Drug Standards (WS_3_-B-3825-98-2012), containing 0.5 g safflower per mL.

### 2.2. Chemicals

Normal human plasma was supplied by Biology Institute of Shanxi (Shanxi, China). The PT kit was produced by Taiyuan Boaote Biotech Co., Ltd. (Shanxi, China). The APTT kit was purchased from Shanghai SunBio Medical Biotechnology Co., Ltd. (Shanghai, China). The reference standards of *p*-hydroxybenzaldehyde and *p*-hydroxy-cinnamic acid were obtained from Tianjin Yifang Science & Technology Co., Ltd. (Tianjin, China) with purity > 98.0%. Ligustrazine hydrochloride injection was purchased from Chengdu Brilliant Pharmaceutical Group (Chengdu, China). Acetonitrile of high performance liquid chromatography (HPLC) grade was purchased from Fisher Scientific Inc. (Pittsburgh, PA, USA). Normal saline (0.9%) was purchased from Henan Kelun Pharmaceutical Co., Ltd. (Henan, China). The other reagents were all of analytical grade and water was double-distilled water.

### 2.3. Anticoagulant Activity Screening of Various Safflower Injections 

To evaluate the anticoagulant activity (APTT and PT) of various safflower injections, some preparations were conducted as follows. Safflower injections were diluted with normal saline (NS) to create sample test solutions at five different concentrations: 100%, 85%, 72%, 61%, and 52%, with an inter-solution ratio of 0.85. Normal human plasma supplied by Biology Institute of Shanxi was anticoagulated with 3.8% sodium citrate (9:1, *v*/*v*) and centrifuged at 2500 rpm for 10 min to obtain platelet-poor plasma (PPP). The anticoagulant (APTT and PT) tests referenced in the literature [16,17] were modified.

#### 2.3.1. APTT Assay of Safflower Injections

APTT reagent was incubated at room temperature (25 °C) and stirred before being used. Calcium chloride (CaCl_2_) solution was incubated at 37 °C for 20 min before being used. APTT was measured using the Sysmex CA-50 Semi-automated Coagulation Analyzer (Sysmex Corporation, Hyogo, Japan). Briefly, the PPP of 50 μL was incubated at 37 °C for 1 min with 50 μL of various concentrations of safflower injections. NS and ligustrazine hydrochloride injection (LHII) (50 μL each) were added separately into equal volumes of PPP to create the blank and positive control groups, respectively. Then, we waited 3 min after adding 50 μL of the APTT reagent. The APTT assay was initiated as soon as 50 μL CaCl_2_ (25 mmol/L) was added. The mixture was continuously monitored until clot formation was recorded automatically. Each sample was measured three times in parallel, and the results were recorded. Graphs of coagulation time (s) against the concentration of safflower injection were plotted to determine the change in coagulation time with the variation in the concentration of safflower injections produced by seven different pharmaceutical companies.

#### 2.3.2. PT Assay of Safflower Injections

PT reagent was incubated at 37 °C for 10 min before use. PT was measured using the Semi-automated Coagulation Analyzer. Briefly, the PPP of 30 μL was incubated at 37 °C for 3 min with 20 μL of various concentrations of safflower injections, and 20 μL of NS and LHII were added separately into equal volumes of PPP as the blank and the positive control groups, respectively. The PT assay was initiated as soon as 100 μL of PT reagents were added. The mixture was continuously monitored, and the time of the clot formation was automatically recorded. Each sample was measured three times in parallel and the results were recorded. 

### 2.4. Preparation of Chemical Fractions in Safflower Injections

The preparation was conducted with CHEETAH^TM^ MP Series Preparative Chromatography System (Tianjin Bonna-Agela Technologies Co., Ltd., Tianjin, China). The safflower injection (1000 mL) (Huawei Pharmaceutical Co., Ltd., Taiyuan, China, Batch 16062020), containing approximately 500 g of safflower, was filtered by vacuum pump, and the filtrate was collected for testing. D101 macroporous resin (650 g) (Cangzhou Baoen Chemical Co., Ltd., Cangzhou, China), soaked and washed with 95% ethanol, was used to fill the LC column using the wet method, with a diameter/height ratio of 1:10. The column was then rinsed using double-distilled water until no ethanol was present. For each preparation, 200 mL of safflower injection was injected into the columns following pre-column, which were then sequentially eluted by water, 20%, 40%, and 60% ethanol. The eluents were collected, vacuum-concentrated, freeze-dried, and four chemical component groups were obtained: Fr.1, Fr.2, Fr.3, and Fr.4.

### 2.5. APTT Assay of Four Chemical Component Groups

The APTT of the four chemical fractions were assessed according to the method stated in Section 2.3.1. Among them, Fr.1, Fr.2, and Fr.3 were dissolved in NS, whereas Fr.4 was dissolved in dimethylsulfoxide (DMSO)-containing NS solution, with DMSO accounting for 1.3% of the evaluation system, to obtain sample test solutions with a concentration of 12.5, 25.0, 50.0, and 100.0 μg/μL corresponding to 0.5, 1.0, 2.0, and 4.0 μg/μL, respectively, in the experimental system. Each test required the addition of 44 μL of PPP and 6 μL of sample test solutions, as well as the blank group and the positive control group. Each sample was assessed three times in parallel and the results were recorded.

### 2.6. Analysis, Separation, and Identification of the Active Ingredient Groups 

HPLC analysis was performed using an Agilent 1260 Infinity Quaternary LC System (Agilent Technologies Inc., Santa Clara, CA, USA). The conditions of HPLC analysis [18] for the active fractions were set as follows: Venusil XBP C18 (2) column (250 mm × 4.6 mm, 5 μm) (Tianjin Bonna-Agela Technologies Co., Ltd., Tianjin, China) and acetonitrile (A), 0.5% phosphoric acid aqueous solution (B), with a linear gradient elution of 5–11% A at 0–10 min, 11–14% A at 10–16 min, 14% A at 16–23 min, 14–20% A at 23–30 min, and 20–35% A at 30–70 min. The flow rate was 1.0 mL/min, and the sample injection column was 10 μL. The samples were detected with a 280-nm wavelength and a temperature of 35 °C. The analysis conditions of qualitative ultra-high performance liquid chromatography mass spectrometry (UHPLC-MS) for the main chemical fraction were set as follows: ACQUITY UPLC^®^ HHS C18 column (2.1 mm × 100 mm, 1.8 μm) (Waters, Milford, MA, USA), and 0.1% formic acid acetonitrile (A), 0.1% aqueous formic acid (B) with a linear gradient elution of 5–10% A at 0–3 min, 10–13% A at 3–5 min, 13–45% A at 5–10 min, 45% A at 10–13 min, 45–-50% A at 13–15 min, 50–70% A at 15–18 min, and 70–95% A at 18–20 min. The flow rate was 0.2 mL/min, and the sample injection column was 5 μL. The samples were detected with a 280-nm wavelength and temperature of 40 °C.

The chemical structures of these components were confirmed by either referring to the reference substances or by using the hydrogen nuclear magnetic resonance (^1^H-NMR) and carbon NMR (^13^C-NMR) spectroscopy techniques following repeated column chromatographic purification using Sephadex LH-20 (GE Healthcare Inc., Piscataway, NJ, USA) and C18 column (9.4 mm × 250 mm, 5 μm) (Agilent Technologies Inc., Santa Clara, CA, USA). 

### 2.7. APTT Assay of Chemical Components 

The APTT of the various chemical components were assessed according to the method stated in Section 2.5. All chemical components were dissolved in DMSO-containing NS solution, with DMSO accounting for 1.3% of the assessment system. 

### 2.8. Statistical Analysis

All the data obtained were expressed as mean ± S.D. (*n* = 3). All statistical analyses of the data were performed by the independent-sample *t*-test, and *p* < 0.05 (vs. control group) was considered statistically significant.

## 3. Results and Discussion

### 3.1. Anticoagulant Activity of Safflower Injection

#### Effects of Safflower Injection on Plasma APTT and PT

The study was initially conducted by evaluating the effect of safflower injections on prolongation of the APTT and PT of normal human plasma in vitro. 

**Figure 1:**
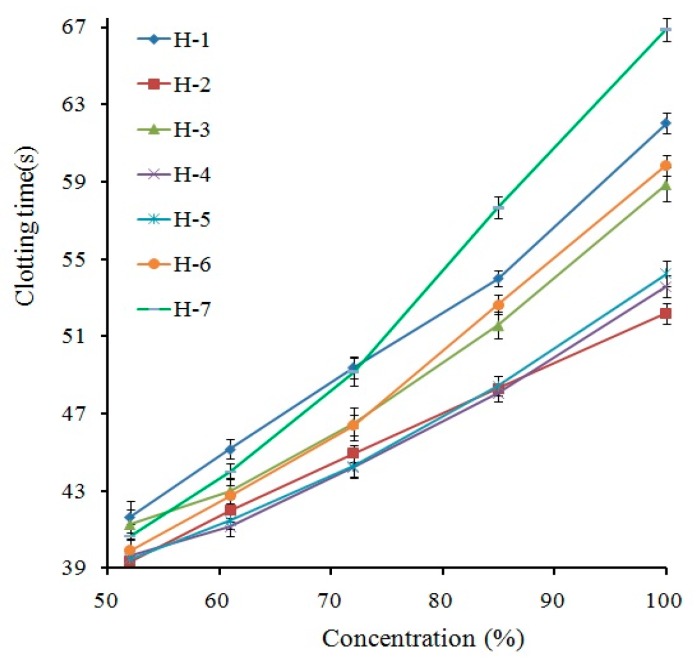
Activated partial thromboplastin time (APTT) effects of safflower injections produced by different companies. The clotting time is expressed as the mean of three measurements ± SD (*n* = 3). Safflower injections were diluted with normal saline (NS) to create sample test solutions at five different concentrations: 100%, 85%, 72%, 61%, and 52%, with an inter-solution ratio of 0.85. H1–H7 denote seven different safflower injections produced by different companies.

The APTT of the safflower injection was evaluated using the dose-effect graphs as shown in Figure 1, comparing the coagulation time with the concentration of the injections. The result indicated that APTT increased with increasing concentration. Taking H-6 as an example, as the concentration of the injection increased from 52% to 100%, the APTT lengthened from 39.7 s to 59.2 s, which was considered a significant prolonging effect (*p* < 0.05 vs. control group). In addition, all safflower injections produced by different companies considerably prolonged APTT, and the result was consistent with that published by Wang et al. [15]. Therefore, safflower injections were suggested to be able to effectively regulate intrinsic coagulation pathways [19]. In addition, according to Figure 1, the APTT of safflower injection from different companies was different under the same concentration. The reasons for this difference might be due to the different sources of safflower used by the seven companies, which caused the different content of the main active components. Moreover, some differences in the control of production process parameters could also lead to the same result.

The PT test was also applied to measure the anticoagulant activity of safflower injections. However, when the concentration of the safflower injection increased, the increase in PT (shown in Table S1) was insignificant (*p* > 0.05 vs. control group). Therefore, in the next experiment, the effects of various chemical fractions and components in safflower injections on PT were not investigated. 

The results showed that safflower injections significantly affect the prolonging of APTT, but insignificantly affect the prolonging of PT. The safflower injection could effectively improve the intrinsic coagulation system, which may be one of the mechanisms for its efficacy in promoting blood circulation [20,21].

### 3.2. Effects of Various Chemical Fractions on Plasma APTT

A representative sample (Huawei Pharmaceutical Co., Ltd., Taiyuan, China, Batch 16062020), whose APTT value was an intermediate value of all, was chosen for further separation and activity screening, and its fractionation could be applied to all samples. The sample was fractionated by D101 macroporous resin with ethanol-water gradient elution, and the fractions were marked as Fr.1, Fr.2, Fr.3, and Fr.4. The anticoagulant activity of each fraction was determined using the APTT test. As shown in Table 1, all four fractions effectively prolonged APTT. When the concentration reached 1.0 μg/μL, the prolonging effects of Fr.3 and Fr.4 were significant (*p* < 0.05 vs. control group). Compared to the blank group, when the concentration reached 4.0 μg/μL, the APTTs of Fr.3 and Fr.4 increased by 37.5 s and 56.3 s, respectively. The trend in the APTT-prolonging effect of the four fractions was as follows, in descending order: Fr.4, Fr.3, Fr.2, then Fr.1. Based on this result, we deduced that Fr.4 is the main active fraction in safflower injections. In short, Fr.4 showed the best anticoagulant activity, since it significantly prolonged APTT at a lower concentration.

### 3.3. Structural Analysis of the Main Chemical Components in Fr.4

**Figure 2:**
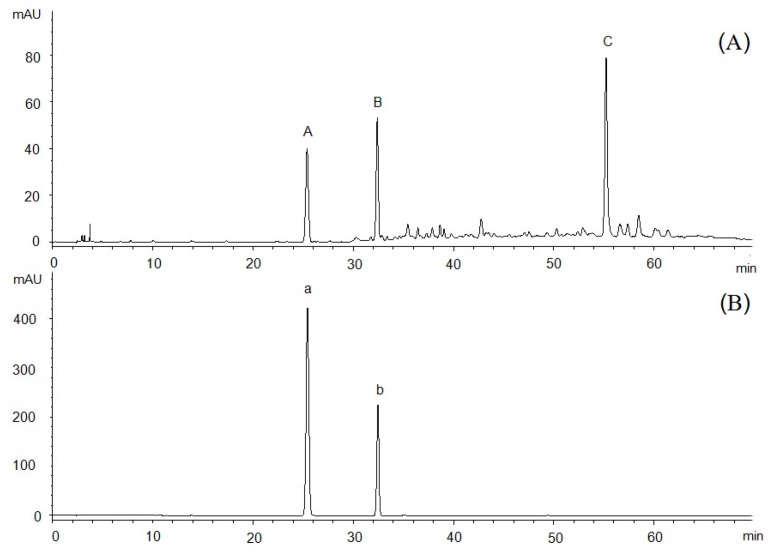
High-performance liquid chromatography (HPLC) chromatograms of fraction Fr.4 and reference substances. (**A**) Chromatograms of Fr.4 and (**B**) chromatograms of reference substances. On chromatogram B of the reference substances “a” is *p*-hydroxybenzaldehyde and “b” is *p*-hydroxy-cinnamic acid.

The HPLC chromatogram of Fr.4 is shown in Figure 2A. After assessment by different wavelengths, peaks A, B, and C were found to be the most dominant chromatographic peaks. Based on UHPLC-MS, peak A yielded a deprotonated ion at 121 *m*/*z* [M − H]^−^, whereas that of peak B was 163 *m*/*z* [M − H]^−^.Peak C formed fragment ions at 311 *m*/*z* [M + H]^+^ and 333 [M + Na]^+^. By comparing the retention times and mass spectrum with reference compounds, we identified that peak A corresponded to *p*-hydroxybenzaldehyde and peak B to *p*-hydroxy-cinnamic acid (chromatograms omitted here). 

**Figure 3:**
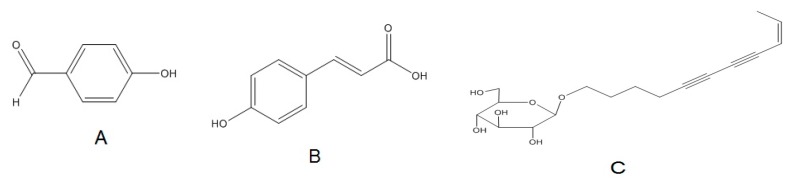
Structures of (**A**) *p*-hydroxybenzaldehyde; (**B**) *p*-hydroxy-cinnamic acid; and (**C**) (8*Z*)-decaene-4,6-diyne-1-*O*-β-d-glucopyranoside.

The compound of peak C was then sequentially purified using Sephadex LH-20, with an eluent consisting of chloroform and methanol in a ratio of 1:1, and a C18 column with an isocratic elution with 77% water and 23% acetonitrile. Eight milligrams of a pale-yellow oily substance was obtained, with the following characterization: ^1^H-NMR (CDCl_3_, 600 MHz) δ: 6.15 (1H, dq, *J* = 10.8, 6.6 Hz, H-9), 5.52 (1H, d, *J* = 10.8 Hz, H-8), 4.36 (1H, d, *J* = 7.5 Hz, H-1′), 3.97-3.35 (6H, saccharide ring H), 3.56 (2H, overlapped, H-1), 2.5 (2H, t, *J* = 6.0 Hz, H-3), 1.93 (3H, dd, *J* = 6.6, 1.2 Hz, CH_3_-10), 1.89 (2H, m, *J* = 6.6 Hz, H-2); ^13^C-NMR (CDCl_3_, 150 MHz) δ: 68.7 (CH_2_, C-1), 28.1 (CH_2_, C-2), 15.6 (CH_2_, C-3), 65.6 (C, C-5), 72.2 (C, C-7), 78.5 (C, C-6), 84.1 (C, C-4), 109.1 (CH, C-8), 142.5 (CH, C-9), 16.3 (CH_3_, C-10), 102.8 (CH, C-1′), 73.4 (CH, C-2′), 77.2 (CH, C-3′), 69.3 (CH, C-4′), 76.2 (CH, C-5′), and 61.5 (CH_2_, C-6′). The data were consistent with the data reported by Zhao et al. [14]. Therefore, peak C was identified as (8*Z*)-decaene-4,6-diyne-1-*O*-β-d-glucopyranoside (Figure 3C).

### 3.4. Effects of the Main Chemical Components on Plasma APTT

Having the strongest APTT activity, Fr.4 was further separated and purified to determine the active components using Sephadex LH-20 and C18 column chromatography. The compounds A, B, and C were isolated. The effects of compound A, B, and C on plasma APTT are shown in Table 2. Compared to the blank control, compounds A and B significantly prolonged APTT. When the concentration was 1.0 μg/μL, the APTTs of compounds A and B were significantly prolonged by 14.7 s and 66.7 s, respectively. Although the APTT was only 57.3 s (prolonged by 18.2 s) at a concentration 4.0 μg/μL, compound C also prolonged APTT. Therefore, we deduced that the three compounds, *p*-hydroxybenzaldehyde, *p*-hydroxy-cinnamic acid, and (8*Z*)-decaene-4, 6-diyne-1-*O*-β-d-glucopyranoside, are the effective ingredients that prolong APTT. 

## 4. Conclusions

In conclusion, safflower injections significantly prolonged APTT in normal human plasma in vitro, but had no obvious influence on PT. Safflower injections could effectively improve the intrinsic coagulation system, and this improvement may be one of the mechanisms for its efficacy in promoting blood circulation in dissipating blood stasis. In addition, we demonstrated that *p*-hydroxybenzaldehyde, *p*-hydroxy-cinnamic acid and (8*Z*)-decaene-4,6-diyne-1-*O*-β-d-glucopyranoside were the effective ingredients in safflower injections by prolonging APTT.

## Figures and Tables

**Table 1:** Activated thromboplastin time (APTT) of fractions Fr.1, Fr.2, Fr.3, and Fr.4. Values are expressed as the mean of three measurements ± SD (*n* = 3). Concentration is the concentration of the sample in the testing system.

Concentration (μg/μL)	APTT (s)				
	LHII	Fr.1	Fr.2	Fr.3	Fr.4
Control Group	34.0 ± 0.4	34.0 ± 0.4	34.0 ± 0.4	34.0 ± 0.4	39.1 ± 0.4
0.8	64.6 ± 0.7 ***	-	-	-	-
0.5	-	35.9 ± 0.8	35.7 ± 0.5	35.3 ± 0.2	39.6 ± 0.4
1.0	-	36.6 ± 0.5	36.0 ± 0.5	39.2 ± 0.8 **	41.1 ± 0.5 *
2.0	-	37.5 ± 1.0	38.0 ± 0.7 **	47.6 ± 0.5 ***	51.4 ± 0.5 ***
4.0	-	37.7 ± 0.1 *	40.0 ± 0.5 **	71.5 ± 0.9 ***	95.4 ± 1.4 **

When compared with the control group, * denotes *p* < 0.05; ** denotes 0.001 < *p* < 0.01; *** denotes *p* < 0.001, and - denotes unevaluated.

**Table 2:** Effects of compounds A, B, and C on plasma APTT. Values are expressed as mean of three measurements ± SD (*n* = 3).

Sample	Concentration (μg/μL)	APTT (s)
Control group	-	39.1 ± 0.4
LHII	0.8	64.6 ± 0.7 ***
A	0.5	46.9 ± 0.8 *
	1.0	53.8 ± 1.0 **
	2.0	72.6 ± 0.9 ***
	4.0	>200.0 ***
B	0.5	53.8 ± 0.8 **
	1.0	105.8 ± 0.8 ***
	1.2	158.3 ± 4.7 ***
C	2.0	40.7 ± 0.2
	4.0	57.3 ± 0.7 ***

Concentration, *, **, and *** have the same meanings as in Table 1. “A” refers to *p*-hydroxybenzaldehyde, “B” refers to *p*-hydroxy-cinnamic acid, and “C” refers to (8*Z*)-decaene-4,6-diyne-1-*O*-β-d-glucopyranoside.

## Supplementary Materials

The following are available online.
