# Supplementary material for: In Vitro Anticoagulant Activity and Active Components of Safflower Injection
Source: Molecules. 2018 Jan 15;23(1):170. doi: 10.3390/molecules23010170 (PMC6017571; doi:10.3390/molecules23010170)
Supplement: Supplementary file 1 [file molecules-23-00170-s001.pdf]

Tittle: In Vitro Anticoagulant Activity and Active Components of Safflower Injection

Supplementary materials

Table S1. Prothromboplastin time（PT）of different companies of Safflower Injection ($\bar{x}$±s, *n* = 3)

PT /s	percentage composition /%		
	52%	71%	100%
Control group		15.13 ± 0.9	
H-1	15.7 ± 0.5	16.7 ± 0.1	17.3 ± 0.2
H-2	15.9 ± 0.2	16.0 ± 0.3	17.6 ± 1.2
H-3	16.1 ± 0.2	16.3 ± 0.3	17.2 ± 0.1
H-4	16.1 ± 0.5	17.3 ± 0.7	18.5 ± 0.3
H-5	16.8 ± 0.4	17.9 ± 0.6	18.4 ± 0.4
H-6	16.4 ± 0.3	17.6 ± 0.7	18.8 ± 0.6
H-7	16.2 ± 0.1	16.6 ± 0.2	18.0 ± 0.3
